# Benz­yl(phen­yl)phosphinic acid

**DOI:** 10.1107/S1600536811008245

**Published:** 2011-04-07

**Authors:** Robert A. Burrow, Rubia M. Siqueira da Silva

**Affiliations:** a Laboratório de Materiais Inorgânicos, Universidade Federal de Santa Maria, Av. Roraima, 1000 Camobi, 97105-900 Santa Maria, RS, Brazil

## Abstract

The title compound, C_13_H_13_O_2_P, crystallized as enanti­omerically pure crystals; for the crystal measured, the P atom has *R* stereochemistry. The crystal structure displays O—H⋯O hydrogen bonding, which links individual mol­ecules related by a 2_1_ screw axis parallel to the crystallographic *a*-axis direction into continuous chains.

## Related literature

For background to phosphinic acids, see: Beckmann *et al.* (2009 ▶); Burrow *et al.* (2000 ▶); Chen & Suslick (1993 ▶); Siqueira *et al.* (2006 ▶); Vioux *et al.* (2004 ▶). For a description of the Cambridge Structural Database, see: Allen (2002 ▶). Geom­etrical analysis was performed with *Mogul* (Bruno *et al.*, 2004 ▶).
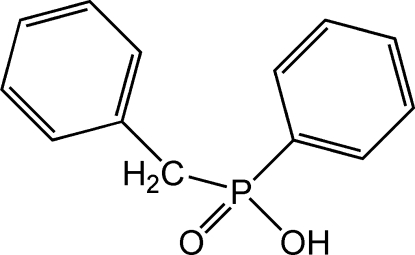


## Experimental

### 

#### Crystal data

C_13_H_13_O_2_P
*M* *_r_* = 232.20
Orthorhombic, 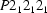

*a* = 5.7326 (2) Å
*b* = 12.3430 (3) Å
*c* = 16.7794 (4) Å
*V* = 1187.27 (6) Å^3^
*Z* = 4
Mo *K*α radiation
μ = 0.21 mm^−1^
*T* = 295 K
0.65 × 0.34 × 0.22 mm

#### Data collection

Bruker X8 Kappa APEXII diffractometer
Absorption correction: gaussian (*SADABS*; Bruker, 2009 ▶) *T*_min_ = 0.880, *T*_max_ = 0.964
14337 measured reflections
3451 independent reflections
3119 reflections with *I* > 2σ(*I*)
*R*_int_ = 0.028

#### Refinement

*R*[*F* ^2^ > 2σ(*F* ^2^)] = 0.039
*wR*(*F* ^2^) = 0.108
*S* = 1.05
3451 reflections
148 parameters
H atoms treated by a mixture of independent and constrained refinement
Δρ_max_ = 0.47 e Å^−3^
Δρ_min_ = −0.23 e Å^−3^
Absolute structure: Flack (1983 ▶), 1447 Friedel pairs
Flack parameter: 0.00 (11)

### 

Data collection: *APEX2* (Bruker, 2009 ▶); cell refinement: *SAINT* (Bruker, 2009 ▶); data reduction: *SAINT* (Bruker, 2009 ▶); program(s) used to solve structure: *SHELXS97* (Sheldrick, 2008 ▶); program(s) used to refine structure: *SHELXL97* (Sheldrick, 2008 ▶); molecular graphics: *DIAMOND* (Brandenburg, 2009 ▶); software used to prepare material for publication: *publCIF* (Westrip, 2010 ▶).

## Supplementary Material

Crystal structure: contains datablocks I, global. DOI: 10.1107/S1600536811008245/zb2011sup1.cif

Structure factors: contains datablocks I. DOI: 10.1107/S1600536811008245/zb2011Isup2.hkl

Additional supplementary materials:  crystallographic information; 3D view; checkCIF report

## Figures and Tables

**Table 1:** Hydrogen-bond geometry (Å, °)

*D*—H⋯*A*	*D*—H	H⋯*A*	*D*⋯*A*	*D*—H⋯*A*
O1—H1⋯O2^i^	0.93 (3)	1.58 (3)	2.4838 (18)	163 (3)

Symmetry code: (i) 
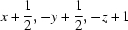
.

**Fig. 1.**
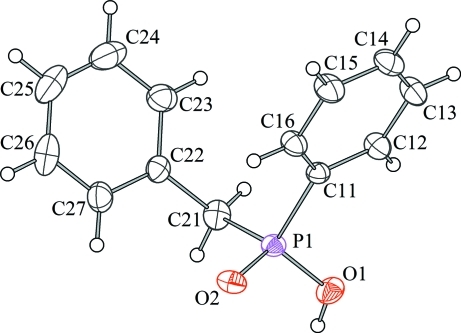
The molecular of (I) structure of the title compound, showing 30% probability ellipsoids.

**Fig. 2.**
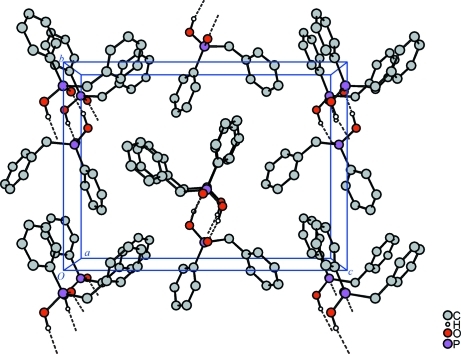
The packing diagram of (I) in the crystallographic a direction with the crystallographic b axis pointing up. The O—H···O intermolecular hydrogen bond is shown dashed.

## supplementary crystallographic information

### Comment

Phosphinic acids have found use for the construction of coordination polymers [Siqueira *et al.*, 2006; Beckmann *et al.*, 2009] for a wide range of applications [Vioux *et al.*, 2004; Chen & Suslick,1993]. Continuing our research on phosphinic acids [Burrow *et al.*, 2000], we report the synthesis and crystal structure of the title compound, (I).

The crystal structure of (I) is from an enantiomerically pure crystal (Flack parameter = 0.00 (11); 1447 Friedel pairs; Flack & Bernardinelli, 2000] with the P atom possessing *R* stereochemistry. An analysis of the geometry of (I) by *Mogul* [Bruno *et al.*, 2004] using the CSD. [Allen, 2002] shows no unusual features for the benzyl and phenyl groups. However, an unusually long P═O bond [P1═O2 = 1.5104 (13) Å; average in *Mogul*:1.484 (17) Å for 16 observations, |*z* score| = 1.568] and a wider C—P—C angle [C11—P1—C21 angle = 109.493 (9)°; average in *Mogul*: 106.9(2.0) °, |*z* score| = 1.281] are found.

The individual molecules of (I) related by a 2_1_ screw axis parallel to the crystallographic *a* direction are joined by hydrogen bonding of the type OH···O=P—OH···O=P to form continuous chains. The short P—O···O=P distance of 2.4838 (18) Å indicates a strong hydrogen bond. This is slightly shorter than the average O···O interaction distance in the CSD. [2.51 (5) Å, 45 observations] for other phosphinic acids.

### Experimental

To a solution of phenylphosphinic acid (2.0 g, 14.1 mmol) in dichloromethane, 30 ml diisopropylethylamine (5.16 ml, 29.6 mmol) and trimethylsilyl chloride (3.74 ml, 29.6 mmol) were separately added at 0 °C under argon. The reaction mixture was stirred at room temperature for 2–3 h, cooled to 0 °C and 1-(bromomethyl)benzene (1.84 ml, 15.5 mmol) was added. After further stirring at room temperature for 48 h, the solvent was removed under vacuum. The residue was suspended in hydrochloric acid (2 *M*, 20 ml) and filtered on a glass frit. The white solid was washed with acetone and dried giving a yield of 1.70 g (65%) of pure product. IR: 1494 (*m*), 1439 (*s*), 1242 (*m*), 1132 (*versus*), 1069 (*s*), 969 (*versus*), 845 (*s*), 787 (*s*), 751 (*s*), 734 (*s*), 701 (*s*), 585 (*m*), 524 (*s*), 477 (*s*), 466 (*m*) cm^-1^. TGA: 310–361 °C: 99% loss. DTA: 181–193 °C & 310–361 °C endothermic peaks. Crystals suitable for single-crystal X-ray analysis were grown from an acetone solution in a desiccator with silical gel.

### Refinement

The H atom on O1 was found in the difference Fourier map and its position was allowed to refine freely while its isotropic displacement factor was set to 1.5 times that of O1. The H atoms were positioned geometretically and allowed to ride on their parent atoms, with C—H bond lengths of 0.93 Å (aromatic CH) and 0.97 Å (methylene CH_2_) and isotropic displacement parameters equal to 1.2 times *U*_eq_ of the parent atom.

### Crystal data

**Table:**

C_13_H_13_O_2_P	*D*_x_ = 1.299 Mg m^−^^3^
*M**_r_* = 232.20	Melting point = 454–456 K
Orthorhombic, *P*2_1_2_1_2_1_	Mo *K*α radiation, λ = 0.71073 Å
Hall symbol: P 2ac 2ab	Cell parameters from 4929 reflections
*a* = 5.7326 (2) Å	θ = 3.3–28.0°
*b* = 12.3430 (3) Å	µ = 0.21 mm^−^^1^
*c* = 16.7794 (4) Å	*T* = 295 K
*V* = 1187.27 (6) Å^3^	Block, colourless
*Z* = 4	0.65 × 0.34 × 0.22 mm
*F*(000) = 488	

### Data collection

**Table:**

Bruker X8 Kappa APEXII diffractometer	3451 independent reflections
Radiation source: fine-focus sealed tube	3119 reflections with *I* > 2σ(*I*)
graphite	*R*_int_ = 0.028
Detector resolution: 0.0833 pixels mm^-1^	θ_max_ = 30.0°, θ_min_ = 3.5°
φ and ω scans	*h* = −8→8
Absorption correction: gaussian (*SADABS*; Bruker, 2009)	*k* = −17→17
*T*_min_ = 0.880, *T*_max_ = 0.964	*l* = −20→23
14337 measured reflections	

### Refinement

**Table:**

Refinement on *F*^2^	Secondary atom site location: difference Fourier map
Least-squares matrix: full	Hydrogen site location: inferred from neighbouring sites
*R*[*F*^2^ > 2σ(*F*^2^)] = 0.039	H atoms treated by a mixture of independent and constrained refinement
*wR*(*F*^2^) = 0.108	*w* = 1/[σ^2^(*F*_o_^2^) + (0.0579*P*)^2^ + 0.2054*P*] where *P* = (*F*_o_^2^ + 2*F*_c_^2^)/3
*S* = 1.05	(Δ/σ)_max_ = 0.001
3451 reflections	Δρ_max_ = 0.47 e Å^−^^3^
148 parameters	Δρ_min_ = −0.23 e Å^−^^3^
0 restraints	Absolute structure: Flack & Bernardinelli (2000), 1447 Friedel pairs
Primary atom site location: structure-invariant direct methods	Flack parameter: 0.00 (11)

### Special details

**Table:**

Experimental. SADABS (Bruker, 2009) was used to perform the numeric absorption correction based on the crystal dimensions determined by face indexing.The number of Friedel pairs measured is 1447. The crystal was not cut to size as it tended to fracture.
Geometry. All e.s.d.'s (except the e.s.d. in the dihedral angle between two l.s. planes) are estimated using the full covariance matrix. The cell e.s.d.'s are taken into account individually in the estimation of e.s.d.'s in distances, angles and torsion angles; correlations between e.s.d.'s in cell parameters are only used when they are defined by crystal symmetry. An approximate (isotropic) treatment of cell e.s.d.'s is used for estimating e.s.d.'s involving l.s. planes.
Refinement. Refinement of *F*^2^ against ALL reflections. The weighted *R*-factor *wR* and goodness of fit *S* are based on *F*^2^, conventional *R*-factors *R* are based on *F*, with *F* set to zero for negative *F*^2^. The threshold expression of *F*^2^ > σ(*F*^2^) is used only for calculating *R*-factors(gt) *etc*. and is not relevant to the choice of reflections for refinement. *R*-factors based on *F*^2^ are statistically about twice as large as those based on *F*, and *R*- factors based on ALL data will be even larger.

### Fractional atomic coordinates and isotropic or equivalent isotropic displacement parameters (Å^2^)

**Table:**

	*x*	*y*	*z*	*U*_iso_*/*U*_eq_	
C11	0.0173 (3)	0.51865 (12)	0.54568 (9)	0.0333 (3)	
C12	0.2219 (4)	0.53887 (16)	0.58763 (12)	0.0445 (4)	
H12	0.3342	0.4849	0.5926	0.053*	
C13	0.2585 (4)	0.64005 (18)	0.62216 (13)	0.0530 (5)	
H13	0.3935	0.6530	0.6513	0.064*	
C14	0.0955 (4)	0.72087 (17)	0.61322 (14)	0.0539 (5)	
H14	0.1215	0.7886	0.6359	0.065*	
C15	−0.1073 (4)	0.70186 (17)	0.57065 (15)	0.0539 (5)	
H15	−0.2170	0.7567	0.5646	0.065*	
C16	−0.1464 (3)	0.60086 (15)	0.53698 (12)	0.0428 (4)	
H16	−0.2828	0.5881	0.5085	0.051*	
C21	0.1121 (4)	0.37609 (16)	0.41002 (11)	0.0463 (4)	
H21A	0.2768	0.3904	0.4181	0.056*	
H21B	0.0971	0.3022	0.3910	0.056*	
C22	0.0212 (3)	0.45208 (15)	0.34658 (10)	0.0400 (4)	
C23	0.1432 (5)	0.54488 (18)	0.32764 (13)	0.0561 (5)	
H23	0.2820	0.5606	0.3539	0.067*	
C24	0.0593 (6)	0.6153 (2)	0.26926 (16)	0.0756 (8)	
H24	0.1425	0.6777	0.2566	0.091*	
C25	−0.1438 (6)	0.5929 (3)	0.23093 (16)	0.0769 (9)	
H25	−0.2001	0.6403	0.1924	0.092*	
C26	−0.2657 (6)	0.5010 (3)	0.24868 (14)	0.0720 (7)	
H26	−0.4033	0.4855	0.2216	0.086*	
C27	−0.1855 (4)	0.4311 (2)	0.30663 (12)	0.0535 (5)	
H27	−0.2709	0.3693	0.3190	0.064*	
H1	0.101 (5)	0.234 (3)	0.5458 (19)	0.080*	
O1	0.0790 (3)	0.30554 (11)	0.56144 (9)	0.0480 (3)	
O2	−0.2947 (2)	0.37242 (9)	0.49255 (9)	0.0444 (3)	
P1	−0.03542 (8)	0.38743 (3)	0.50410 (3)	0.03482 (11)	

### Atomic displacement parameters (Å^2^)

**Table:**

	*U*^11^	*U*^22^	*U*^33^	*U*^12^	*U*^13^	*U*^23^
C11	0.0375 (8)	0.0272 (6)	0.0350 (7)	−0.0003 (6)	0.0014 (6)	−0.0005 (5)
C12	0.0399 (9)	0.0415 (9)	0.0520 (10)	0.0028 (8)	−0.0048 (8)	−0.0043 (8)
C13	0.0490 (11)	0.0526 (11)	0.0574 (11)	−0.0089 (9)	−0.0107 (9)	−0.0098 (9)
C14	0.0639 (14)	0.0378 (9)	0.0600 (12)	−0.0056 (9)	−0.0009 (10)	−0.0138 (9)
C15	0.0590 (13)	0.0342 (9)	0.0684 (13)	0.0105 (9)	−0.0095 (10)	−0.0101 (9)
C16	0.0420 (9)	0.0341 (8)	0.0525 (10)	0.0031 (7)	−0.0078 (8)	−0.0058 (7)
C21	0.0504 (10)	0.0418 (9)	0.0465 (9)	0.0155 (8)	0.0003 (8)	−0.0070 (8)
C22	0.0468 (10)	0.0405 (8)	0.0327 (7)	0.0060 (7)	0.0035 (7)	−0.0051 (6)
C23	0.0652 (14)	0.0509 (11)	0.0523 (11)	−0.0075 (10)	0.0080 (10)	−0.0044 (9)
C24	0.111 (2)	0.0522 (13)	0.0638 (14)	−0.0030 (16)	0.0222 (15)	0.0105 (12)
C25	0.109 (2)	0.0749 (18)	0.0464 (12)	0.0311 (18)	0.0081 (14)	0.0147 (12)
C26	0.0729 (15)	0.103 (2)	0.0404 (10)	0.0214 (15)	−0.0098 (11)	−0.0021 (12)
C27	0.0559 (12)	0.0641 (13)	0.0405 (10)	−0.0047 (10)	−0.0024 (9)	−0.0037 (9)
O1	0.0649 (10)	0.0328 (6)	0.0461 (7)	0.0112 (6)	−0.0006 (6)	0.0035 (5)
O2	0.0413 (6)	0.0326 (5)	0.0592 (8)	−0.0041 (5)	0.0011 (6)	−0.0015 (6)
P1	0.0409 (2)	0.02614 (17)	0.03746 (19)	0.00195 (14)	0.00110 (17)	−0.00116 (16)

### Geometric parameters (Å, °)

**Table:**

C11—C16	1.390 (2)	C21—H21B	0.9700
C11—C12	1.391 (3)	C22—C23	1.379 (3)
C11—P1	1.7893 (16)	C22—C27	1.386 (3)
C12—C13	1.393 (3)	C23—C24	1.395 (4)
C12—H12	0.9300	C23—H23	0.9300
C13—C14	1.375 (3)	C24—C25	1.359 (5)
C13—H13	0.9300	C24—H24	0.9300
C14—C15	1.384 (3)	C25—C26	1.365 (5)
C14—H14	0.9300	C25—H25	0.9300
C15—C16	1.387 (3)	C26—C27	1.379 (4)
C15—H15	0.9300	C26—H26	0.9300
C16—H16	0.9300	C27—H27	0.9300
C21—C22	1.511 (3)	O1—P1	1.5420 (14)
C21—P1	1.7962 (19)	O1—H1	0.93 (3)
C21—H21A	0.9700	O2—P1	1.5104 (13)
			
C16—C11—C12	119.46 (16)	C23—C22—C21	120.2 (2)
C16—C11—P1	120.38 (13)	C27—C22—C21	121.29 (19)
C12—C11—P1	120.15 (13)	C22—C23—C24	120.3 (3)
C11—C12—C13	119.89 (18)	C22—C23—H23	119.9
C11—C12—H12	120.1	C24—C23—H23	119.9
C13—C12—H12	120.1	C25—C24—C23	120.0 (3)
C14—C13—C12	120.20 (19)	C25—C24—H24	120.0
C14—C13—H13	119.9	C23—C24—H24	120.0
C12—C13—H13	119.9	C24—C25—C26	120.3 (2)
C13—C14—C15	120.26 (18)	C24—C25—H25	119.8
C13—C14—H14	119.9	C26—C25—H25	119.8
C15—C14—H14	119.9	C25—C26—C27	120.2 (3)
C14—C15—C16	119.90 (19)	C25—C26—H26	119.9
C14—C15—H15	120.0	C27—C26—H26	119.9
C16—C15—H15	120.0	C26—C27—C22	120.6 (2)
C15—C16—C11	120.27 (18)	C26—C27—H27	119.7
C15—C16—H16	119.9	C22—C27—H27	119.7
C11—C16—H16	119.9	P1—O1—H1	120 (2)
C22—C21—P1	114.11 (12)	O2—P1—O1	114.73 (8)
C22—C21—H21A	108.7	O2—P1—C11	109.11 (8)
P1—C21—H21A	108.7	O1—P1—C11	106.16 (8)
C22—C21—H21B	108.7	O2—P1—C21	109.93 (9)
P1—C21—H21B	108.7	O1—P1—C21	107.28 (8)
H21A—C21—H21B	107.6	C11—P1—C21	109.49 (9)
C23—C22—C27	118.5 (2)		
			
C16—C11—C12—C13	1.6 (3)	C24—C25—C26—C27	1.1 (4)
P1—C11—C12—C13	−177.83 (16)	C25—C26—C27—C22	−1.1 (4)
C11—C12—C13—C14	−1.6 (3)	C23—C22—C27—C26	0.7 (3)
C12—C13—C14—C15	0.7 (4)	C21—C22—C27—C26	−179.8 (2)
C13—C14—C15—C16	0.1 (4)	C16—C11—P1—O2	−22.31 (17)
C14—C15—C16—C11	−0.1 (3)	C12—C11—P1—O2	157.10 (14)
C12—C11—C16—C15	−0.7 (3)	C16—C11—P1—O1	−146.45 (15)
P1—C11—C16—C15	178.69 (17)	C12—C11—P1—O1	32.96 (17)
P1—C21—C22—C23	102.5 (2)	C16—C11—P1—C21	98.04 (16)
P1—C21—C22—C27	−77.0 (2)	C12—C11—P1—C21	−82.55 (16)
C27—C22—C23—C24	−0.2 (3)	C22—C21—P1—O2	54.98 (17)
C21—C22—C23—C24	−179.8 (2)	C22—C21—P1—O1	−179.66 (14)
C22—C23—C24—C25	0.2 (4)	C22—C21—P1—C11	−64.87 (17)
C23—C24—C25—C26	−0.6 (4)		

### Hydrogen-bond geometry (Å, °)

**Table:**

*D*—H···*A*	*D*—H	H···*A*	*D*···*A*	*D*—H···*A*
O1—H1···O2^i^	0.93 (3)	1.58 (3)	2.4838 (18)	163 (3)

Symmetry codes: (i) *x*+1/2, −*y*+1/2, −*z*+1.
